# Small insects actively respond to updrafts by modulating their wing and body kinematics

**DOI:** 10.1093/icb/icag140

**Published:** 2026-08-03

**Authors:** Evan J Williams, Ignazio Maria Viola, Laura Ross, Katy Monteith, Zhehong Zhang, Robert Baird, David W Murphy

**Affiliations:** Department of Mechanical Engineering, University of South Florida, Tampa, FL 33620, USA; School of Engineering, Institute of Energy Systems, University of Edinburgh, Edinburgh EH9 3FB, UK; School of Biological Sciences, Institute of Ecology and Evolution, University of Edinburgh, Edinburgh EH9 3FL, UK; School of Biological Sciences, Institute of Ecology and Evolution, University of Edinburgh, Edinburgh EH9 3FL, UK; School of Engineering, Institute of Energy Systems, University of Edinburgh, Edinburgh EH9 3FB, UK; School of Biological Sciences, Institute of Ecology and Evolution, University of Edinburgh, Edinburgh EH9 3FL, UK; Department of Mechanical Engineering, University of South Florida, Tampa, FL 33620, USA

## Abstract

Small insects utilize atmospheric flows to facilitate appetitive and migratory behaviors. In the convective and nocturnal boundary layers, these insects may experience high-speed updrafts which can present significant aerodynamic challenges. While it is known from field observations that small insects resist these updrafts, how they do so while maintaining flight stability is unknown. Here we investigate the flight behavior of two insect species, the 3-mm fruit fly (*Drosophila melanogaster*) and the 2-mm fungus gnat (*Lycoriella ingenua*), while exposed to quiescent air and to a steady 0.433 m s^−1^ upward flow within a vertical wind tunnel. We used high-speed 3D photogrammetry to capture flight trajectories and wing and body kinematics. Both species maintained a constant vertical flight velocity in quiescent conditions and in the updraft by maintaining a consistent beat frequency while significantly reducing their stroke amplitude (and thus saving energy), though with some size-specific differences. The smaller fungus gnats kept the dorsal portion of their stroke more so than the larger fruit flies, thereby maintaining the lift-generating clap-and-fling mechanism which is generally required at the lower Reynolds numbers at which they operate. Both species also maintained a similar relative flow velocity, which combines wing tangential velocity and animal velocity with respect to the free stream. Finally, the updraft somewhat affected flight stability in the fungus gnat (as seen in its more sinuous flight trajectories) but not in the fruit fly, which did not change the sinuosity of its trajectories in the updraft. These findings provide a bridge between large- and small-scale studies of small insects in the atmosphere.

## Introduction

Insects encounter a wide variety of complex environmental flows that may significantly challenge or possibly benefit their foraging and dispersal behaviors (Taylor 1974). These environmental flows can be generalized into several categories. First, the flight boundary layer occurs close to the ground (approximately 0–15 m) and has wind speeds that do not exceed insect flight speeds (Taylor 1974). In addition, daytime surface heating generates large-scale updrafts and downdrafts (with speeds of the order of 1 m s^−1^) which comprise the convective boundary layer (CBL). The CBL is part of the atmospheric boundary layer (ABL), which is the portion of the atmosphere that is directly influenced by the Earth’s surface. The ABL has a thickness of approximately 1 km during the day and 100–200 m at night and possesses wind speeds that greatly exceed insect flight speed capabilities (Chapman et al. 2011). Insects performing daytime ascents from the ground are thought to benefit from updrafts within the CBL to reach higher altitudes (Wainwright et al. 2017). In contrast, insects ascending at dusk do not benefit from updrafts as the CBL has been replaced by the more stable nocturnal boundary layer (NBL) and the overlaying residual layer, regions in which horizontal winds aid long-range insect migrations. Even within the NBL, however, insects may also experience updrafts and downdrafts owing to various types of waves or large-scale convergence and divergence within the atmosphere (Wainwright et al. 2020).

A wide variety of insect taxa of various sizes have been found at high altitudes using nets deployed from balloons, towers, kites, and aircraft (Taylor 1974; Chapman et al. 2004). These include representatives from Hemiptera, Diptera, Hymenoptera, and Coleoptera and range in size from sub-mm English grain aphids (*Sitobion avenae*) to noctuid moths several cm in size (Chapman et al. 2016). Recent studies using co-located Doppler lidar and radar systems have allowed simultaneous measurement of the vertical speed of both the background flow and individual insects, thus providing insight into how insects respond to updrafts and downdrafts. For example, Geerts and Mao (2005) found that, during the day, small insects in an updraft resisted the upward flow to slow their ascent relative to the ground but descended in a downdraft. Further, the magnitude of their resistance to the updraft increased with the updraft velocity such that at updraft speeds of 0.5 and 3 m s^−1^, their velocities relative to the updraft were approximately −0.25 and −0.7 m s^−1^, respectively (Wainwright et al. 2017). At night, however, the insects opposed both the updrafts and downdrafts (both of which had lower speeds than during the day), indicating the insects had a preferred altitude (Wainwright et al. 2020). While these studies and others have illuminated the large-scale flight kinematics of small insects in the ABL, the kinematics producing those behaviors are not well understood owing to the difficulty of simultaneously measuring large-scale flight behaviors and small-scale wing and body kinematics in the field. For example, Wainwright et al. (2017) could only speculate that small insects used a type of “energy saving hovering” in a daytime updraft. Conversely, high-speed cameras suitable for laboratory studies provide wing and body kinematics at millimeter or centimeter scales but are often limited to hovering or short translational flights. Thus, a knowledge gap exists between the macro and micro scales of tiny insect flight in the atmosphere and ultimately raises questions of how complex and large-scale flows affect flight dynamics.

Specialized flow facilities provide a strategy to examine insect flight behaviors in quasirealistic atmospheric flows (Visser 1976; Miller and Roelofs 1978; Alexander 1986; Albertani et al. 2013; Fuller et al. 2014). For example, Ortega-Jiménez and Combes (2018) tested the effect of a vertically oriented Rayleigh-Bénard convection cell (with maximum updraft speeds of 0.3 m s^−1^ and turbulence intensities in the range of 24–29%) on fruit fly flight. These authors found that the flies exposed to convection had higher body pitch angles and beat frequences, lower flight speeds, and more sinuous paths. Further, flies which successfully navigated the convection cell flew faster and decreased wing stroke amplitude in comparison to those which failed. These behavioral alterations likely increased flight control and stability in this turbulent environment. Further, various researchers have exposed flying insects to numerous types of turbulence and wind gusts within horizontally situated wind tunnels. For example, hawkmoths (body length of approximately 4.5 cm) responded to a von Karman vortex street in a horizontal wind tunnel with larger body oscillations and an increased wingbeat frequency (Ortega-Jimenez et al. 2013). Further, bumblebees in a horizontal wind tunnel showed little kinematic variability in a head wind, but in a tail wind they rapidly pitched their bodies to control their flight speed (Combes et al. 2023). Finally, while some researchers previously examined insect flight in a vertical wind tunnel to analyze flight endurance (Blackmer and Byrne 1993; Byrne 1999), take-off capabilities (Blackmer and Phelan 1991), and odor tracking (Daykin 1967; Notingham and Hardie 1993), their kinematic responses have not been well characterized.

Here we investigate the flight responses of two insect species, *Drosophila melanogaster* (fruit fly) and *Lycoriella ingenua* (fungus gnat), freely flying in a quiescent fluid and in an updraft generated by a vertical wind tunnel. Members of Drosophilidae and Sciaridae (i.e., fungus gnats) are good candidates for this study as both have been found in the CBL at a height of 200 m (Chapman et al. 2004). Further, fruit flies (2–3 mm in size) were chosen because their flight behaviors are well studied (Lehmann and Dickinson 1998; Ortega-Jiménez and Combes 2018; Gu et al. 2020; Zhu and Sun 2020), thus providing ample opportunities for comparison of results. Fungus gnats (1–2 mm) were additionally chosen because their wing apparatus is morphologically similar to that of fruit flies (i.e., the possession of two wings and halteres) but at a smaller aerodynamic scale which approaches the size and Reynolds number range of “tiny” insects. In this more viscous regime, insects generate large lifting forces during the rowing portion of the stroke (Cheng and Sun 2018; Lyu et al. 2019) and heavily rely on wing-wing interactions (e.g., clap and fling) to generate lift (Weis-Fogh 1973; Lehmann and Pick 2007; Lehmann et al. 2021). We can thus compare how the wing and flight kinematics of morphologically similar insects operating at different Reynolds numbers differ in their response to an updraft. We hypothesize that an updraft will act as a supportive force and result in altered wing and body kinematics as a trade-off between lift-production and stability. Overall, this study aims to bridge the gap between ecological observations and laboratory flight studies by providing insight into the kinematic response of small insects to updrafts.

## Materials and Methods

### Experimental setup

**Fig. 1:**
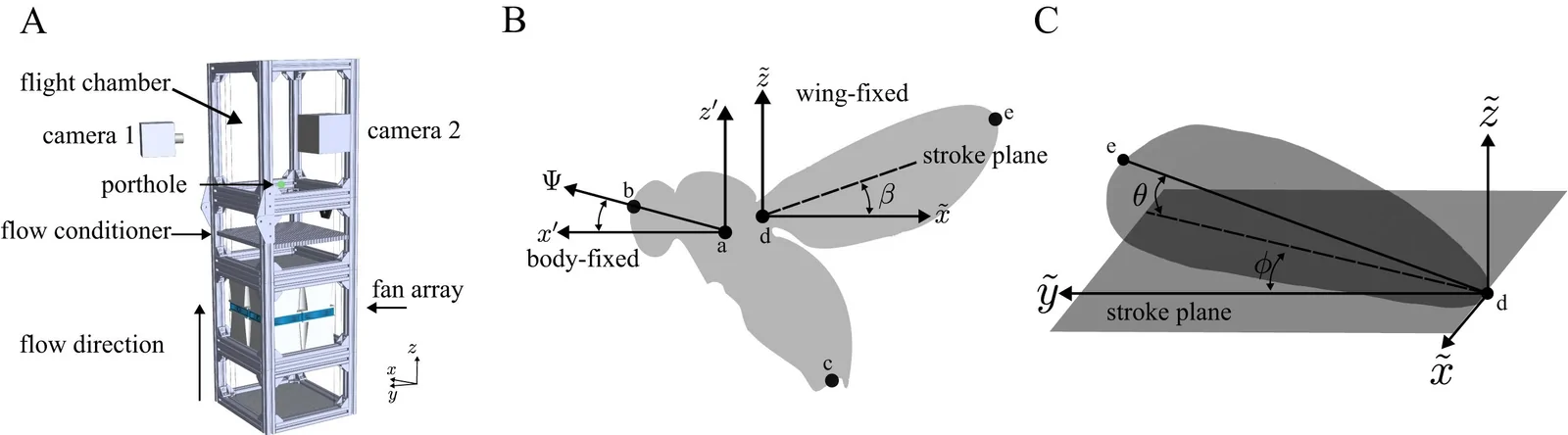
(A) 3D model of the vertical wind tunnel showing the earth-fixed coordinate system (*x,y,z*), camera orientation, and flow direction. (B) Side view of a fruit fly showing the body-fixed ($x^{\prime},\ y^{\prime},\ z^{\prime}$) and wing-fixed ($\tilde{x}$,$\tilde{y}$,$\tilde{z}$) coordinate systems, as well as defining the body angle ${\mathrm{\Psi }}$ and stroke plane angle ${\mathrm{\beta }}$. (C) Insect wing orientation within the wing-fixed coordinate system ($\tilde{x}$,$\tilde{y}$,$\tilde{z}$), where ${\Phi}$ defines the positional angle within the stroke plane and ${\mathrm{\theta }}$ defines the wingtip deviation from the stroke plane.

A 3D photogrammetry system was used to record the wing and body kinematics of freely flying fungus gnats and fruit flies in the working section (250 mm *× * 250 mm *× * 400 mm; *\ x,y,z*) of a vertical wind tunnel, where the updraft is in the positive *z*-direction (Fig. 1A). A 25 mm diameter porthole opening located near the bottom of the wind tunnel allowed insertion of insects before the experiments. Tests were conducted in both a quiescent environment (i.e., with the wind tunnel turned off) and in a vertical updraft with a mean flow speed of *u* = 0.433 m s^−1^, which was the wind tunnel’s maximum flow speed and reasonably close to speeds seen in environmental updrafts. The vertical wind tunnel was characterized in a prior study (Viola et al. 2025) using particle image velocimetry (PIV). The mean velocity across the wind tunnel’s volume is within *± * 2% of its mean value, and the turbulence intensity is less than 1%. Stereophotogrammetric measurements of the insect flight kinematics were made using two orthogonally aligned and temporally synchronized high-speed cameras (Phantom 640 VEO) fitted with 50-mm Nikon macro lenses. These cameras were placed such that the lenses were 65 cm from the wind tunnel’s outer walls. Image acquisition occurred at two different frame rates. Low-speed recordings (at 300 Hz with a spatial resolution of 110 ${\mathrm{\mu m}}$ per pixel within a 181 mm *× * 181 mm *× * 281 mm volume; *x,y,z*) were made to measure large-scale flight trajectories. An additional set of high-speed recordings at a frame rate of 4800 Hz with 110 ${\mathrm{\mu }}$m per pixel spatial resolution with a reduced field of view (112 mm *× * 112 mm *× * 112 mm) were made to measure small-scale wing and body kinematics at high temporal resolution. For both experiments, camera focal planes coincided with the midplane of the wind tunnel. The setup was spatially calibrated using approximately 100 images of a 12.7 cm wand moved and rotated through the common field of view and by applying the direct linear transform technique via the easyWand software (Abdel-Aziz and Karara 1971; Hedrick 2008). The kinematic reconstruction error, evaluated through a bootstrapping method in the DLTdv8 software, was 0.09 mm. This error is a result of balancing the large field of view to quantify flight trajectories while retaining a fine spatial resolution to resolve wing and body kinematics. Uniform backlighting was provided by two illuminator panels, each comprising twelve IR lights (850 nm wide angle 8 ${\mathrm{W}}$, Univivi) arranged in a 4 *× * 3 grid and placed 10 cm from the wind tunnel, and by photography diffusers (40 cm *× * 50 cm, Renian) attached to the wind tunnel’s outer walls.

### Insect collection and husbandry

Fungus gnats were collected from Mycobee Mushroom Farm in North Berwick, UK and maintained in a growth room at 18°C with 70% humidity (Shlyakonova et al. 2026). The fungus gnat cultures were established in April 2023. Successive generations were obtained by introducing several males and females into a new polypropylene plastic vial (25 mm diameter *× * 95 mm height) with a bacteriological agar. Fungus gnats were fed with a powdered mixture of brewer’s yeast, mushroom powder, spinach, nettle powder, and ground straw every 2–3 days until reaching the adult stage (approximately 3 weeks). Males and females possess distinct abdomens and were thus readily identified in the videos. Several generations were yielded from one vial. Similarly, fruit flies from a previously established culture were kept at 24°C in polypropylene plastic vials with a sugar-yeast medium; successive generations occurred in the same vials. All insects were transported from the incubation rooms to the testing area in polypropylene plastic vials. All experiments were conducted in July and August 2023.

### Experimental procedure

All experiments were conducted in a dark room at room temperature (${24}^ \circ $ C) with the computer and operator separated from the test area by a curtain. The experimental temperature was somewhat higher than the culture temperature for fungus gnats, but this difference was previously found to have little effect on fungus gnat behavior (Frouz and Nováková 2001; Shlyakonova et al. 2026). We altered the following parameters systematically across the experimental campaign: wind speed (*u = 0* or $0.443\ {\mathrm{m\ }}{{\mathrm{s}}}^{ - 1}$), frame rate (300 $\ {\mathrm{or\ }}4$800 Hz), and insect species (fungus gnat or fruit fly). Experiments were not randomized as the trials at 300 Hz were all conducted first, followed by the trials at 4800 Hz. For each trial, approximately 150 adult insects (irrespective of sex) were transferred directly from their breeding tubes (50–75 per tube) to the wind tunnel through the porthole opening. We then closed the porthole and set the wind tunnel to the selected wind speed. After a 20-min acclimation period, the experiment began. Throughout the experiment, images from the cameras were actively monitored. The cameras were simultaneously manually triggered using the Phantom software upon observing a flight event. Each trial lasted two hours, after which all insects were removed. No insects were used for multiple trials.

### Kinematic analysis

We used wind tunnel data collected with the larger field of view at 300 Hz to characterize flight trajectories and large-scale body kinematics. Passes were selected for analysis if the insect was in the field of view of both cameras and had a minimum flight time of 0.3 s (i.e., 100 frames). If an insect left the field of view of one camera, the tracking sequence was terminated; any new flight event from a previously terminated track was considered a new pass. We thus analyzed 53 fungus gnat passes (26 at $u = 0{\mathrm{\ m\ }}{{\mathrm{s}}}^{ - 1}$; 27 at $u = 0.443{\mathrm{\ m\ }}{{\mathrm{s}}}^{ - 1}$) and 47 fruit fly passes (22 at $u = 0{\mathrm{\ m\ }}{{\mathrm{s}}}^{ - 1}$; 25 at $u = 0.443{\mathrm{\ m\ }}{{\mathrm{s}}}^{ - 1}$). The fungus gnat and fruit fly passes had mean flight times of approximately 1 and 0.7 ${\mathrm{s}}$, respectively. For the duration of flight, two markerless points, namely the thorax and head (a–b; Fig. 1B) were manually tracked using DLTdv8a (Hedricks 2008), with point a serving as the origin of a body-fixed coordinate system. A third point, the abdomen, was tracked at the first frame of the flight sequence to characterize insect body length ${l}_{\mathrm{b}}$, measured as the distance from the tip of head (point b) to the tip of the abdomen (point c). We also quantified the insect’s body angle ${\mathrm{\Psi }}$, defined as the angle between the body axis (defined as the line connecting points a and b; Fig. 1B) and the horizontal plane (${\mathrm{xy}}$). The animal flight speed *v*, the horizontal flight speed ${v}_{\mathrm{h}}$, and the vertical flight velocity ${v}_{\mathrm{v}}$, all with respect to a lab-fixed frame, were also measured at each time point and averaged across the entire flight sequence. We define the vertical air speed as the animal’s vertical speed with respect to the free stream: ${v}_{\mathrm{a}} = {v}_{\mathrm{v}} - u$. We also define the animal’s relative velocity as ${v}_{{\mathrm{rel}}} = \sqrt {v_{\mathrm{h}}^2 + v_{\mathrm{a}}^2} .$ Here, we note that ${v}_{{\mathrm{rel}}}$ reduces to the flight speed in quiescent flow. Average values of ${\mathrm{\Psi }}$, *v*, ${v}_{\mathrm{h}}$, ${v}_{\mathrm{v}}$, ${v}_{\mathrm{a}}$, and ${v}_{{\mathrm{rel}}}$ for each pass were obtained, and an overall mean value for each experimental condition was then obtained by averaging over all passes. The net-to-gross displacement ratio *η *, which is the ratio of net displacement to flight path length, was also calculated for each pass to characterize path tortuosity. This parameter was calculated for a normalized path length of 100 body lengths for each pass to account for differences in body size between fruit flies and fungus gnats (e.g., Karakas et al. 2020). Finally, fungus gnat sex was determined, with 14 females and 8 males observed in the quiescent case and 9 females and 14 males observed in the upward flow. Fungus gnats are known to have variable sex ratios in culture (Shlyakonova et al. 2026). Sex determination from the images was not possible for fruit flies.

We used data collected with the smaller field of view at 4800 Hz to characterize wing kinematics. Passes were selected for analysis if the insect was near the center of the field of view of both cameras and remained there for five consecutive wing beats. We thus analyzed five videos for each experimental condition. For each pass, the head, both wingtips, and wing root most visible in camera 1 were tracked over these five wing beats (points b, d–e; Fig. 1B and C). A wing-fixed ($\tilde{x},$$\tilde{y},$$\tilde{z}$; Fig. 1C) coordinate system centered on the tracked wing root (Fig. 1B and C) was then established to evaluate the kinematics of the single wing. From these points, the average wing length ${l}_{\mathrm{w}}$ and average wingbeat frequency *f* were measured and the wingtip trajectory as described by the Euler angles ${\mathrm{\beta }}$, ${\Phi}$, and ${\mathrm{\theta }}$ were reconstructed for each pass (Ellington 1984; Williams et al. 2025). Here, the stroke plane angle ${\mathrm{\beta }}$ is defined as shown in Fig. 1B and is determined by performing a linear regression of the wingtip trajectory projected into the $\tilde{y} = 0$ plane (Fig. 1B). To obtain the stroke plane angle, for each individual wing beat we used a least-squares fit line based on the plotted wingtip trajectory. The positional angle ${\Phi}$ describes wingtip position within the stroke plane, with positive values indicating dorsal positions (Fig. 1C). The stroke deviation angle ${\mathrm{\theta }}$ describes out-of-plane motion, with positive values above the stroke plane (Fig. 1C). In addition, we calculated stroke amplitude ${\mathrm{\Phi }} = {\phi}_{{\mathrm{max}}} - {\phi}_{{\mathrm{min}}}$ and stroke deviation amplitude ${\mathrm{\Theta }} = {{\mathrm{\theta }}}_{{\mathrm{max}}} - {{\mathrm{\theta }}}_{{\mathrm{min}}}$. The wing kinematics were calculated for each wing stroke and then averaged to find an individual’s mean response. The local wing-speed Reynolds number $R{e}_{\mathrm{w}} = \bar{c}U/{\mathrm{\nu }}$ was calculated, where $\bar{c}$ is the average chord length, *ν * is the kinematic viscosity of air, and $U = 2{\mathrm{\Phi }}f{r}_{\mathrm{g}}$ is the average wing speed at the radius of gyration ${r}_g = \sqrt {\frac{1}{s}\mathop \smallint \limits_0^R {r}^2c( r )dr} $, where *R* is the length of the wing, *c* is the chord length at a radial distance *r* from the wing root, and *s* is the wing area. Further, to account for the updraft and the insect’s forward flight, we also calculated an effective Reynolds number $R{e}_{{\mathrm{eff}}} = \bar{c}{U}_{{\mathrm{eff}}}/{\mathrm{\nu }}$, where ${U}_{{\mathrm{eff}}} = U + \ {v}_{{\mathrm{rel}}}$ is an approximate estimate of the effective speed combining contributions from flapping, body translation, and the updraft. Due to limited camera resolution, direct measurements of $\bar{c}$ and ${r}_{\mathrm{g}}$ were challenging. Therefore, these parameters were approximated from separate microscopic images of each species (Shin et al. 2012). The following relationships were found for fungus gnats, $\overline {{c}_{{\mathrm{fg}}}} = 0.29{l}_{\mathrm{w}}$;$\ {r}_{g,fg} = 0.67{l}_{\mathrm{w}}$, and fruit flies, $\overline {{c}_{{\mathrm{ff}}}} = 0.34{{\mathrm{l}}}_{\mathrm{w}}$;${\mathrm{\ }}{r}_{{\mathrm{g}},{\mathrm{\ ff}}} = 0.65{l}_{\mathrm{w}}$, and were used to calculate $R{e}_w$ and $R{e}_{eff}$.

### Statistical analysis

For the low-speed recordings, a Shapiro–Wilk test was used to assess normality for each parameter (${p}_{\mathrm{w}}\ > \ 0.\ 05$ indicates that the data do not significantly deviate from normality). For normally distributed parameters, a two-tailed heteroscedastic *t*-test was used to determine statistically significant differences. Similarly, for parameters that were not normally distributed, the median values were compared using a ranked sum Wilcoxon test. For measured parameters, we used a two-staged averaging procedure—first by averaging an individual’s response, followed by averaging across individuals—to compute the mean kinematic response (two-staged averaged parameters are indicated with an overbar). The quiescent and wind cases for each species were then compared using either a two-tailed heteroscedastic *t*-test or a ranked sum Wilcoxon test, depending on the normality of data. Additionally, a two-tailed heteroscedastic *t*-test was used to evaluate responses between sexes in fungus gnats. For each wingbeat, we measured approximately 30 points and 19 points for the fungus gnats and fruit flies, respectively. To account for the slight variation in wingbeat frequency across individuals, the mean wing kinematics were upsampled to fifty points across the stroke period using bicubic interpolation.

To control Type I errors (incorrectly rejecting the null hypothesis), a Holm–Bonferroni correction was applied across both recording frequencies to adjust the initial significance level of *P* = 0.05. For each trial, the parameters were ranked in ascending order based on *P*-values and compared to their respective corrected significant levels. Then each calculated *P*-value was compared to its Holm–Bonferroni corrected *p*-value. The first *P*-value to be greater than its adjusted *P*-value resulted in all subsequent parameters failing to reject their null hypothesis. For the low-speed analysis, four parameters (*η *, $\overline {{v}_{\mathrm{h}}} $, $\overline {{v}_{\mathrm{v}}} $, ${\mathrm{\bar{\Psi }}}$) were analyzed as a response to the updraft which adjusted the *P*-values to the following corrections: ${p}_{{\mathrm{L}}1}$= 0.0125, ${p}_{{\mathrm{L}}2}$= 0.0167, ${p}_{{\mathrm{L}}3}$= 0.0250, and ${p}_{{\mathrm{L}}4}$= 0.05. Similarly, for the high-speed analysis four parameters ($\overline {{f}_{\mathrm{w}}} ,{\mathrm{\ \bar{\beta }}},{\mathrm{\ \bar{\Phi }}},{\mathrm{\ \bar{\Theta }}}$) were analyzed with the following corrections: ${p}_{{\mathrm{H}}1}$= 0.0125, ${p}_{{\mathrm{H}}2}$= 0.0167, ${p}_{{\mathrm{H}}3}$= 0.0250, and ${p}_{{\mathrm{H}}4}$= 0.05.

### Terminal velocity measurements

To determine the level of powered flight required by the insects in the updraft, we measured the terminal velocities of both fruit flies and fungus gnats via drop tests in an enclosed settling column (2.0 m *× * 0.8 m *× * 0.8 m). The column’s height (*h/l ≫ *100, where *l\ *is the insect’s body length) is sufficient to ensure the insects achieved terminal velocity at the observation window. Insects were anesthetized with $C{O}_2$ and handled with tweezers. As a result of the anesthetization, the insect wings were not extended during the drop tests. Videos of falling insects were captured at 100 Hz using a synchronized dual-camera shadowgraphy setup (LaVision Imager sCMOS) with zero stereo angle, positioned opposite an LED-illuminated background. Custom MATLAB algorithms tracked the flyer’s high-contrast silhouette to determine its precise center and orientation. This algorithm used thresholding and Canny edge detection to isolate the animal’s silhouette to accurately track its centroid throughout the test. To minimize errors arising from perspective projection and lens distortion, the terminal velocity was computed by dividing the vertical separation between the cameras' optical axes by the transit time between these two focal planes. Five insects were tested for each species (three females and two males). Here we note that the drop tests occurred in 2026, well after the original *L. ingenua* line was abandoned (and therefore unavailable for the terminal velocity tests). To compensate for the lack of *L. ingenua*, we used the fungus gnat species *Bradysia coprophila*, which has a gross morphology essentially indistinguishable from *L. ingenua*, as a suitable alternative.

## Results

Terminal velocities for the insects with wings not extended were 2.23 *± * 0.121 m s^−1^ and 1.32 *± * 0.0721 m s^−1^ for the fruit flies and fungus gnats, respectively. Terminal velocities with wings fully extended would likely be approximately 50% less (Thomas et al. 1977). Even in this case, the relatively low updraft speed ($u = 0.443{\mathrm{\ m\ }}{{\mathrm{s}}}^{ - 1}$) compared to the measured terminal velocities indicates that the updraft was not sufficient to support the insects at a constant height, thus requiring powered flight.

**Fig. 2:**
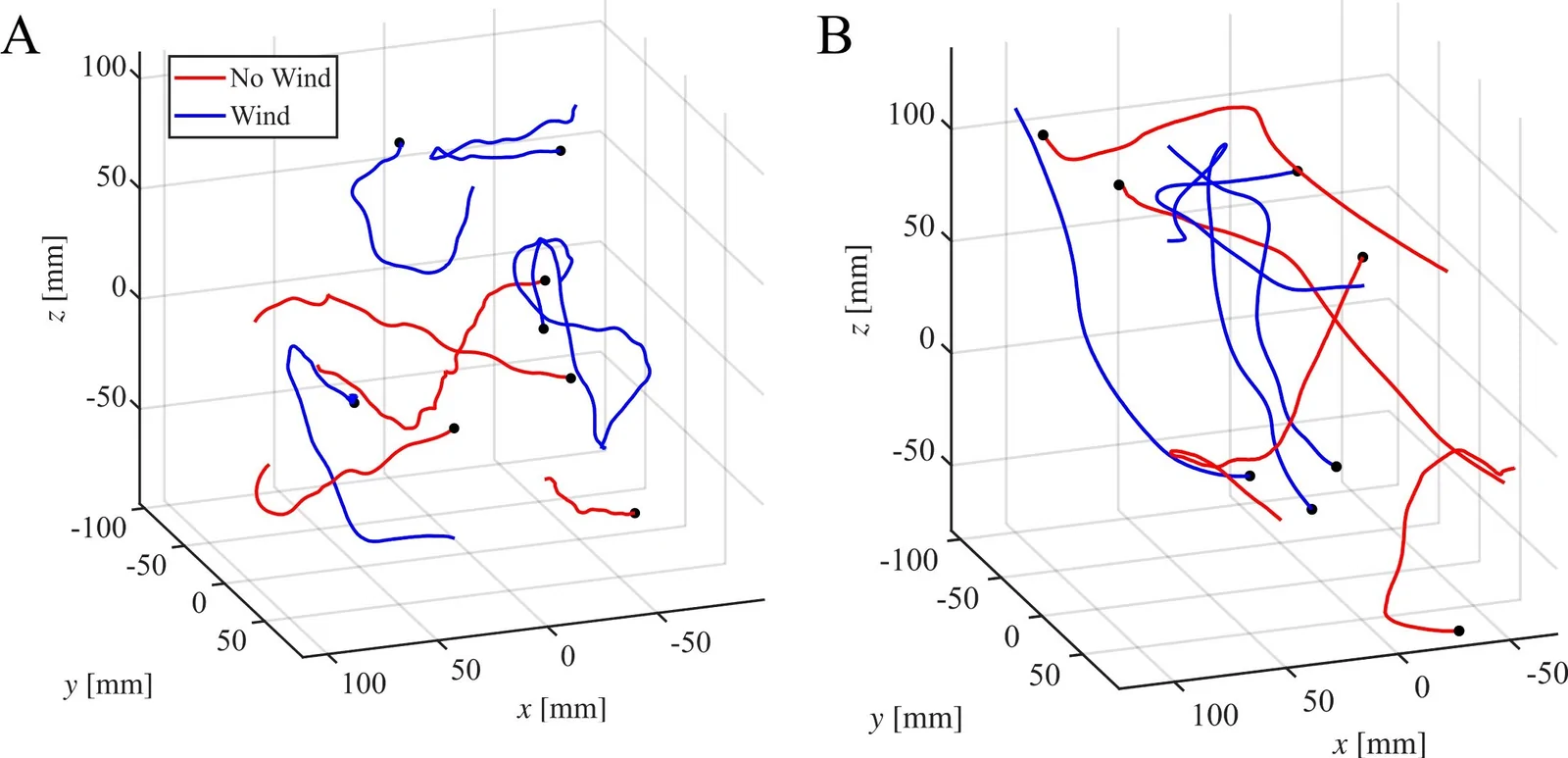
Representative flight trajectories of freely flying insects in an earth-fixed coordinate system. Black dots indicate the beginning points. Red lines represent a quiescent (no wind) environment, and blue lines represent the presence of a vertical wind (i.e., in the positive *z*-direction; ${\boldsymbol{n}} = 4$ for each case). Fungus gnat flight trajectories (A). Fruit fly flight trajectories (B).

We first examine large-scale behaviors obtained while recording at a low frequency (300 Hz) and then examine the wingbeat kinematics recorded at high frequency (4500 Hz). Wind altered flight trajectories differently in fungus gnats and fruit flies (Fig. 2A; Table 1). Fungus gnats flying in a vertical updraft significantly decreased *η * by 32% (*p = 0.0115*), indicating more sinuous flight paths in the updraft as compared to quiescent conditions. In contrast, *η * of the fruit flies did not significantly change as the insects maintained relatively straight flight trajectories in both quiescent conditions and in the updraft. (Fig. 2B; Table 1). We did not find evidence that either species altered their vertical flight velocity ${\bar{v}}_{\mathrm{v}}$ (in the laboratory reference frame) in the vertical updraft when compared to quiescent conditions. This lack of evidence indicates that, similar to field studies (Wainwright et al. 2017, 2020), the insects are attempting to maintain their altitude, possibly by actively reducing their mean lift coefficient. Further, both species significantly decreased their body angle${\mathrm{\ \bar{\Psi }}}$ in the updraft (Table 1), fruit flies by 45% ($p = 4.08\ \times {10}^{ - 5}$) and fungus gnats by 18% $( {p = 7\ \times {{10}}^{ - 6}} )$. This reduced body angle is likely linked to increases in flight speed, which will be discussed subsequently. For fungus gnats, there was no indication that overall flight speed $\bar{v}$ or horizontal flight speed $\overline {{v}_{\mathrm{h}}} \ $changed in the updraft. However, fruit flies in the vertical updraft increased $\bar{v}$ and significantly increased $\overline {{v}_{\mathrm{h}}} \ $(by 68.9%; $p = 5.4\ \times {10}^{ - 4}$). Finally, we found no evidence that male and female fungus gnats differed in body size or kinematic behaviors in either quiescent or updraft conditions.

**Table 1:** Kinematic and body measurements of fungus gnat and fruit fly response in an updraft.

Parameter	No wind	Wind	*P*-value
Fungus gnats			
${\boldsymbol{\eta }}$	$0.812 \pm {\rm}0.25$	$0.545 \pm {\rm}0.35$	0.0115*
$\overline {\boldsymbol{v}} $ (mm s^−1^)	$164.75 \pm {\rm}92.4$	$180.4 \pm {\rm}76.6$	–
$\overline {{{\boldsymbol{v}}}_{{\bf h}}} \ $ (mm s^−1^)	$155.03 \pm {\rm}83.1$	$170.78 \pm {\rm}69.5$	0.20
$\overline {{{\boldsymbol{v}}}_{{\bf v}}} \ $ (mm s^−1^)	$13.17 \pm {\rm}67.6$	$7.48 \pm {\rm}71.4$	0.85
$\overline {{{\boldsymbol{v}}}_{{\bf a}}} \ $ (mm s^−1^)	$13.17 \pm {\rm}67.6$	$- 425.5 \pm {\rm}71.4$	–
$\overline {{{\boldsymbol{v}}}_{{\boldsymbol{rel}}}} \ $ (mm s^−1^)	$164.75 \pm {\rm}92.4$	*453.5 ± 84.9*	–
${{\bf \bar{\Psi }}}\ $ (${{\bf deg}}.$)	$48.22 \pm {\rm}4.11$	$39.58 \pm {\rm}5.69$	$7 \times {10}^{ - 6}$ *
${{\boldsymbol{l}}}_{{\bf b}}$ (mm)	*1.91 ± 0.50*	*1.92 ± 0.23*	–
$\overline {{{\boldsymbol{l}}}_{{\bf w}}} \ $ (mm)	*2.43 ± 0.08*	*2.39 ± 0.22*	–
$\overline {{{\boldsymbol{f}}}_{{\bf w}}} $ (Hz)	$164.8 \pm {\rm}6.97$	$157.4 \pm {\rm}23.7$	0.059
${{\bf \bar{\beta }}}\ $ (deg.)	$12.80 \pm {\rm}18.3$	$- 18.77 \pm {\rm}29.6$	0.084
${{\bf \bar{\Phi }}}\ ( {{{\bf deg}}.} )$	$111.9 \pm {\rm}6.6$	$75.43 \pm {\rm}18.6$	0.008*
${{\bf \bar{\Theta }}}\ ( {{{\bf deg}}.} )$	*22.78 ± 3.41*	$23.16 \pm {\rm}2.36$	0.469
$\overline {{\boldsymbol{R}}{{\boldsymbol{e}}}_{{\bf w}}} \ $	*50.0 ± 4.4*	*28.65 ± 8.96*	–
$\overline {{\boldsymbol{R}}{{\boldsymbol{e}}}_{{\boldsymbol{eff}}}} \ $	*55.3 ± 3.69*	*56.36 ± 11.1*	–
Fruit flies			
${\boldsymbol{\eta }}$	$0.695 \pm {\rm}0.21$	0.751$\ \pm {\mathrm{\ }}0.19$	0.292
$\overline {\boldsymbol{v}}$ (mm s^−1^)	$276.4{\rm} \pm {\rm}149$	$474.1 \pm {\rm}123$	–
$\overline {{{\boldsymbol{v}}}_{{\bf h}}} \ $ (mm s^−1^)	$245.00 \pm {\rm}143$	*414.70 ± 135*	$5.4 \times {10}^{ - 4}$ *
$\overline {{{\boldsymbol{v}}}_{{\bf v}}} \ $ (mm s^−1^)	$6.22 \pm {\rm}108$	$- 10.44 \pm {\rm}227$	0.71
$\overline {{{\boldsymbol{v}}}_{{\bf a}}} \ $ (mm s^−1^)	$6.22 \pm {\rm}108$	$- 443.4 \pm {\rm}227$	–
$\overline {{{\boldsymbol{v}}}_{{\boldsymbol{rel}}}} \ $ (mm s^−1^)	$276.4{\rm} \pm {\rm}149$	*624 ± 217*	–
${{\bf \bar{\Psi }}}\ $ (${{\bf deg}}.$)	$37.68 \pm {\rm}5.33$	$20.04 \pm {\rm}14.1$	$4.08 \times {10}^{ - 5}$ *
${{\boldsymbol{l}}}_{{\bf b}}$ (mm)	*2.38 ± 0.08*	*2.42 ± 0.06*	–
$\overline {{{\boldsymbol{l}}}_{{\bf w}}} \ $ (mm)	*2.26 ± 0.16*	*2.28 ± 0.18*	–
$\overline {{{\boldsymbol{f}}}_{{\bf w}}} $ (Hz)	$243.2{\rm} \pm {\rm}16.4$	237.9 *± * 9.9	0.555
${{\bf \bar{\beta }}}\ $ (deg.)	−1.62 *± *16.1	6.04 *± *12.0	0.469
${{\bf \bar{\Phi }}}\ ( {{{\bf deg}}.} )$	113.07 *± * 17.8	78.12 *± *12.5	0.0085*
${{\bf \bar{\Theta }}}\ ( {{{\bf deg}}.} )$	24.4 *± *3.50	26.34 *± *3.70	0.42
$\overline {{\boldsymbol{R}}{{\boldsymbol{e}}}_{{\bf w}}} \ $	*76 ± 14.2*	*55 ± 12.8*	–
$\overline {{\boldsymbol{R}}{{\boldsymbol{e}}}_{{\boldsymbol{eff}}}} \ $	*95.8 ± 21.1*	*91.00 ± 17.6*	–

*Notes: P*-values marked with an asterisk indicate statistical significance after the Holm–Bonferroni correction. Parameters with an overbar were averaged in two stages.

**Fig. 3:**
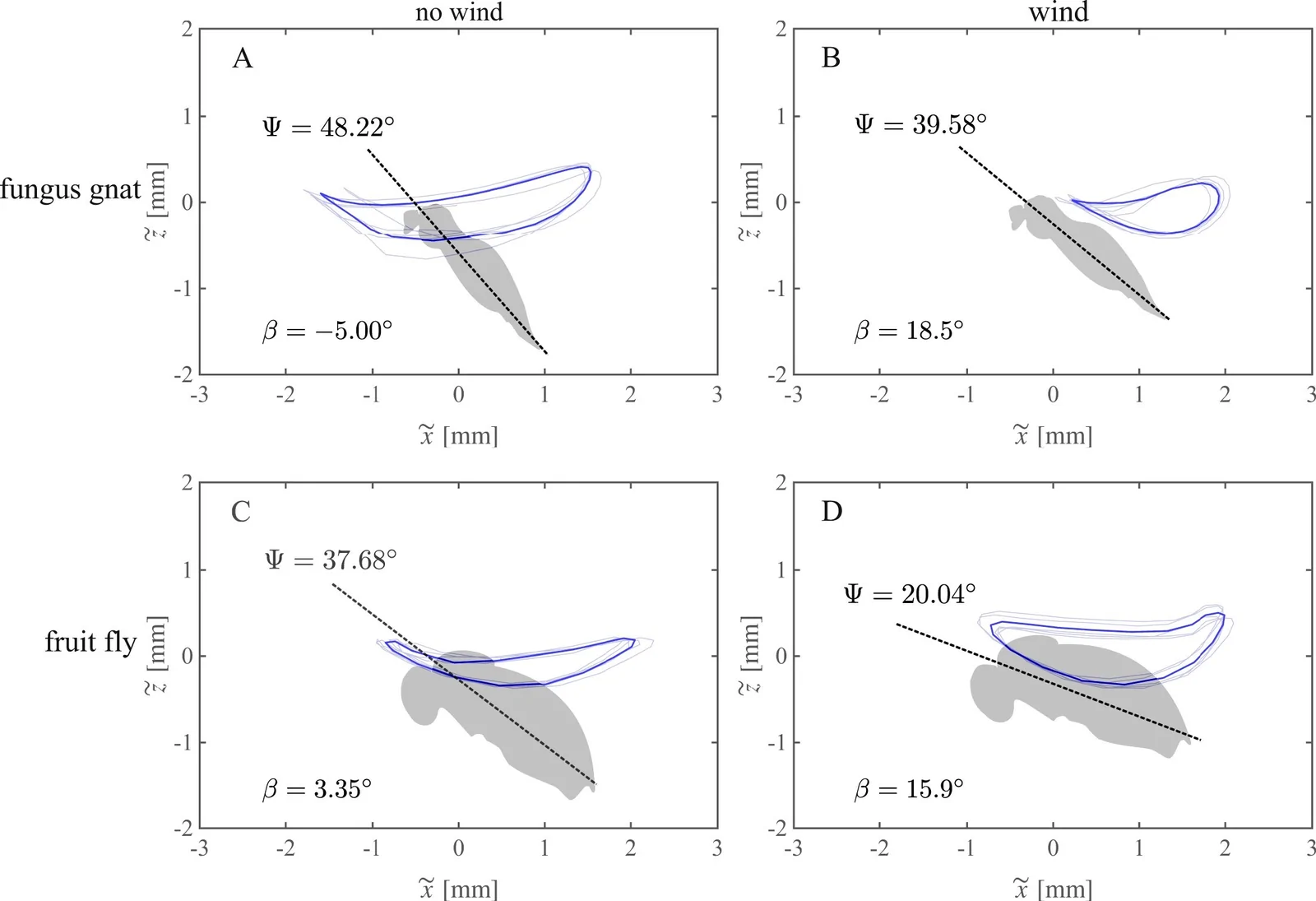
A comparison of wingtip trajectories of fungus gnats and fruit flies in quiescent conditions and vertical wind. Each case represents a single individual’s response across five wingbeats. The bold blue and light blue lines represent an insect’s average and individual wingtip trajectories, respectively. The insect silhouettes are oriented by their respective ${{\bf \Psi }}$. The origin represents the insect’s wing root. Each panel represents a species response to the vertical wind: (A and B) fungus gnats and (C and D) fruit flies.

**Fig. 4:**
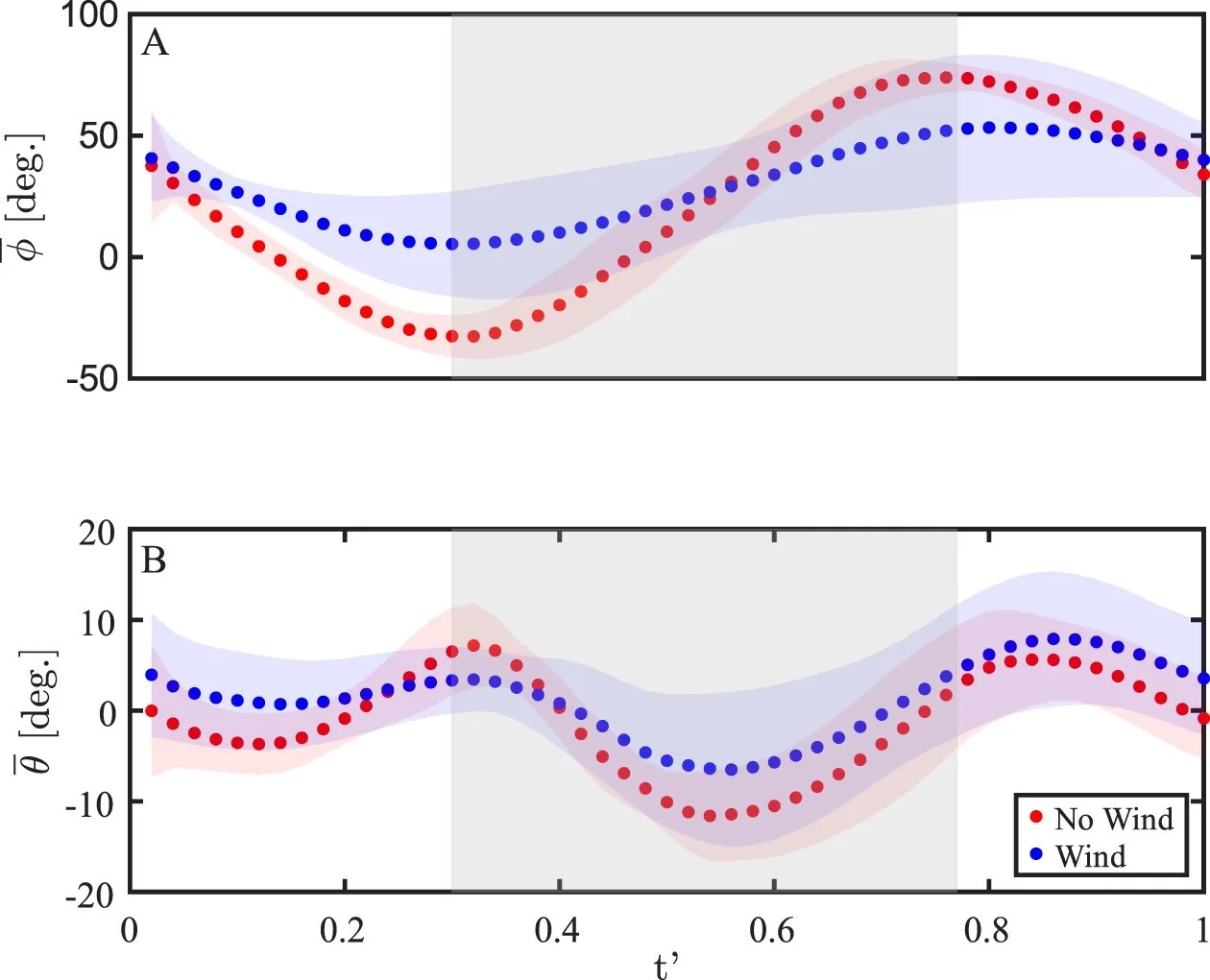
Fungus gnat wing kinematic responses to a vertical wind. Average fungus wing kinematics freely flying in no wind (*u = 0* m s^-1^; red) and in a vertical wind (*u = 0*.433 m s^-1^; blue). Time $t\mathrm{'}$ is nondimensionalized by the average wingbeat period for each species and flow scenario (Table 1). Blue- and red-shaded areas represent the standard deviations at each respective time point. The gray-shaded area indicates the rowing portion of the flapping cycle. The measured Euler angles show a comparison of wingtip positional angles ${\Phi}$ (A) and stroke deviation angles ${\mathrm{\theta }}$ (B).

**Fig. 5:**
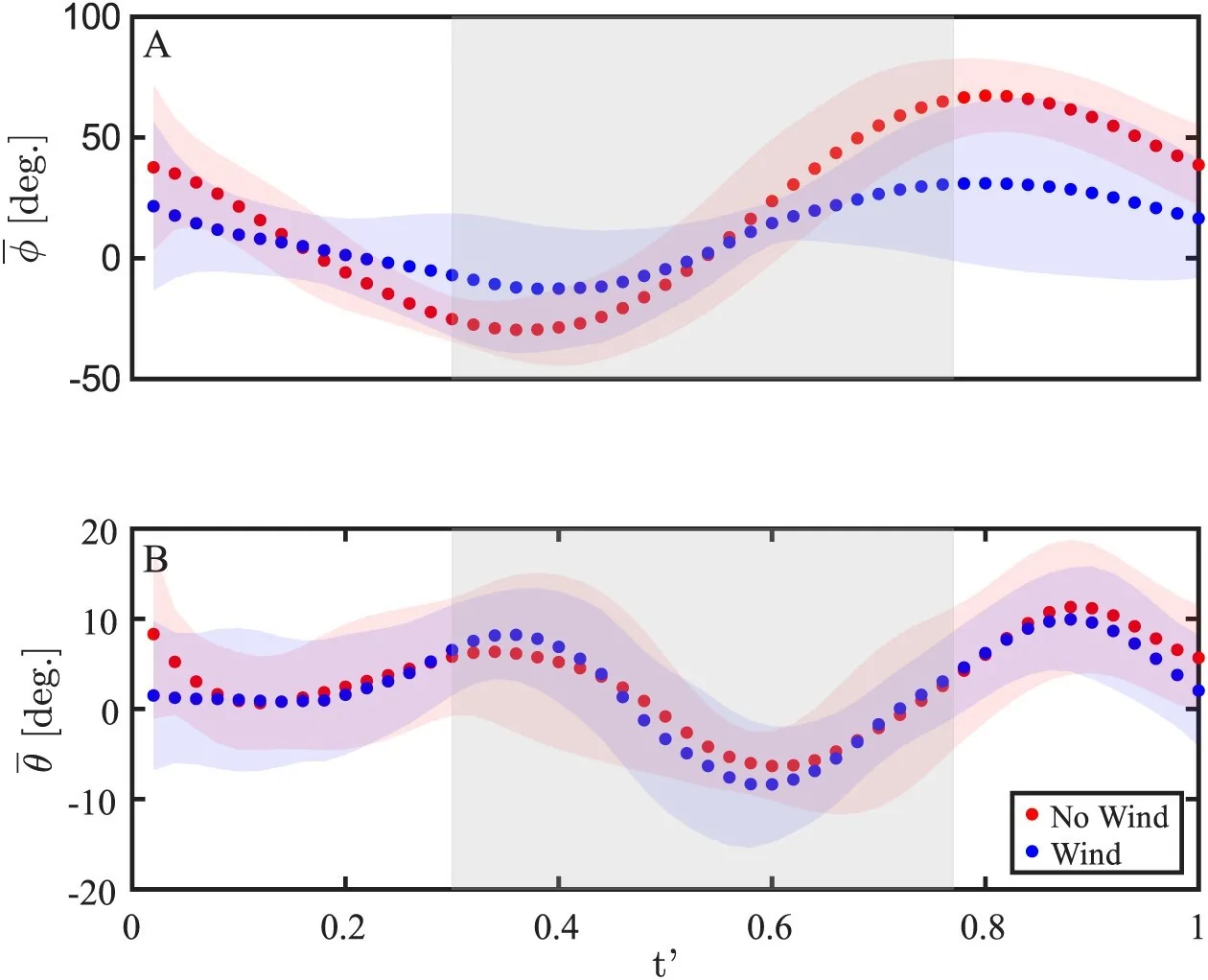
Fruit fly kinematic responses to a vertical wind. Average fungus wing kinematics freely flying in no wind (*u = 0* m s^-1^; red) and in a vertical wind (*u = 0*.433 m s^-1^; blue). Time $t\mathrm{'}$ is nondimensionalized by the average wingbeat period for each species and flow scenario (Table 1). Shaded areas represent the standard deviations at each respective time point. The gray-shaded area indicates the rowing portion of the flapping cycle. The measured Euler angles show a comparison of wingtip positional angles ${\Phi}$ (A) and stroke deviation angles ${\mathrm{\theta }}$ (B).

We now examine results from the high-speed recordings. Individual insects retained relatively similar wing kinematics across all five wingbeats irrespective of flow conditions (Fig. 3A–D; Supplementary Figs. 1 and  2). However, the updraft induced large differences in wing kinematics in both species. While fungus gnats in quiescent conditions exhibited a full wing stroke with the wingtip extending both ventrally and dorsally relative to the wing root, the fungus gnats in the updraft restricted their stroke trajectories both ventrally and dorsally (but more so ventrally) such that the wings remained in mainly dorsal positions, with mean positional angle $\overline {{\Phi}} > 0^\circ $ (Figs 3A and B, and 4A). As a result, fungus gnats in the updraft significantly decreased their stroke amplitude ${\mathrm{\bar{\Phi }}}$ by 32.5% (*P* = 0.008; Table 1). Likewise, the fruit flies restricted the extent of their stroke trajectories, resulting in a significant decrease in their stroke amplitude by 30.9% (Figs. 3C and D, and 5A; Table 1). However, neither species in the updraft presented detectable changes in stroke deviation amplitude ${\mathrm{\bar{\Theta }}},$ stroke plane angle ${\mathrm{\bar{\beta }}}$, or wingbeat frequency $\overline {{f}_{\mathrm{w}}} $ (Table 1). The time series of mean stroke deviation angle was also quite similar in both quiescent conditions and in the updraft for both species (Figs. 4B and 5B). In a vertical wind, the wing-speed Reynolds number $\overline {R{e}_{\mathrm{w}}} $ decreased by 43.1% and 27.6% for the fungus gnats and fruit flies, respectively. Conversely, the effective Reynolds number $\overline {R{e}_{{\mathrm{eff}}}\ } $ yielded similar values irrespective of wind conditions. In a vertical wind, fungus gnats increased $\overline {R{e}_{{\mathrm{eff}}}\ } $ by 1.9% while fruit flies decreased $\overline {R{e}_{{\mathrm{eff}}}\ } $ by 5.0%.

## Discussion

Field studies have long found small insects high above the ground, both within the CBL during the day and in the NBL and residual layer at night (Taylor 1974; Chapman et al. 2004, 2010, 2011, 2016; Reynolds and Reynolds 2009; Reynolds et al. 2017; Wainwright et al. 2017; Wainwright et al. 2020). For example, sub-millimeter whiteflies have been found at an elevation of 40 m, and other species have been found at elevations of over 200 m (Chapman et al. 2004), thus requiring the insects to ascend several thousand body lengths (Byrne et al. 1996; Byrne 1999). These insects may benefit from updrafts within the CBL during the day (Wainwright et al. 2017) and must also deal with updrafts convecting them above their preferred elevation at night (Wainwright et al. 2020). Indeed, Wainwright et al (2017, 2019, 2020) showed in the field that small insects in updrafts, during the day and night, descend with respect to the rising air. However, the method by which the insects achieve this has remained unknown.

Our results in a dark wind tunnel echo these nighttime field results, showing that both fruit flies and fungus gnats successfully resist being displaced upward in an updraft. Our results also reveal for the first time the alterations in wing and body kinematics which effect this resistance. Both fungus gnats and fruit flies significantly reduce their body angle and stroke amplitudes while keeping their beat frequencies constant. This seems to alter the wing kinematics, allowing the insect to maintain altitude and reduce the energetic cost of flight. When the insect moves downwards with respect to the free stream, the flow around the wing is different from that of an insect moving vertically with the free stream (or, equivalently, flying horizontally in quiescent flow). Therefore, to maintain a mean upward force that matches their weight, the animal must adapt its wing kinematics. This study reveals that it does it by reducing body angle and stroke amplitude while keeping frequency constant. These results show similarities with those reported by Zhu and Sun (2020) in a horizontal wind tunnel. They found that, with increasing horizontal wind speed, fruit flies decreased their stroke amplitude up to a speed of approximately 0.7 m s^−1^ while not increasing beat frequency. In contrast, Ortega-Jiménez and Combes (2018) found that fruit flies successfully navigating a convective cell decreased stroke amplitude while slightly increasing their flapping frequency as compared to flying in quiescent flow. This higher beat frequency may stem from the greater turbulence intensity in their facility (25–29%) as compared to our vertical wind tunnel (lower than 1%). Specifically, the fruit flies in their convection cell experience large vortices which may significantly perturb their flight trajectory. Their altered kinematics likely decrease the response time and increase the flight control of the fruit flies, thus allowing them to respond more effectively to aerodynamic perturbations in the convection cell (Ortega-Jiménez and Combes 2018). Another difference between our results and those in the convection cell studied by Ortega-Jiménez and Combes (2018) is that the fruit flies in our updraft significantly increased horizontal flight speed (with a corresponding increase in $\overline {{v}_{{\mathrm{rel}}}} $ to 624 ± 217 mm s^−1^; Table 1) and, as flight speed and body angle are known to be inversely correlated in this species (Zhu and Sun 2020), also decreased their body angle. This coupled behavior, often seen in larger insects (Dudley and Ellington 1990; Willmott and Ellington 1997), likely reduces body drag forces, while simultaneously modulating the thrust vector (Meng and Sun 2016). In contrast, the fruit flies in the convection cell decreased their flight speed and thus increased their body pitch angle (though flies successfully crossing the convection cell had higher flight speeds and lower body angles than those which failed). This reduced flight speed may reflect the fruit flies reacting to the spatially heterogeneous flow in the convection cell as compared to the uniform flow in the vertical wind tunnel.

For the smaller fungus gnats, the flight speed and horizontal flight speed both increased in the updraft by approximately 10% (an insignificant increase in $\overline {{v}_{\mathrm{h}}} )$, with a corresponding increase in $\overline {{v}_{{\mathrm{rel}}}} $ to 453.5 ± 84.9 mm s^−1^; Table 1). The body angle significantly decreased from 48° to 40°. It is not known if speed and body angle are inversely correlated (as in fruit flies), but, as these species are morphologically similar, it seems likely that they are and that the decrease in body angle is due to the increase in horizontal flight speed. Alternative explanations of the shallower body angle include torques generated by the dorsal restriction of the wing stroke and associated changes in the stroke plane angle (for both species), a desire to fly downwards, or an effort to increase the projected body area in the direction of flow (thus modifying the orientation of the resultant aerodynamic force relative to body weight). Regardless, the increased horizontal speed ${v}_{\mathrm{h}}$ of both species may reflect a desire to leave the updraft or may simply be enhanced appetitive flight (e.g., searching for food or mates). Finally, the constant beat frequency of the fungus gnat in quiescent and updraft conditions is in alignment with previous studies of other tiny insects under a different loading condition (Byrne et al. 1988; Wootton 1992; Byrne 1999). For example, submillimeter tobacco whiteflies (*Bemisia tabaci*) did not alter their beat frequency in a flight chamber when exposed to a downward flow (0.16 m s^−1^) and were observed to fly several hours against the flow (Byrne 1999).

Our results also provide insight into how the wing aerodynamics of both species may be altered in an updraft and how variations in the Reynolds number regime for the differently sized species may play a role in this. Remarkably, despite the local wing-speed Reynolds number $\overline {R{e}_w} $ decreasing by 43% and 28% for fruit flies and fungus gnats, respectively, $\overline {R{e}_{{\mathrm{eff}}}} $ remains essentially constant across quiescent and updraft conditions for both species as the reduction in *U* is compensated for by a corresponding increase in ${v}_{{\mathrm{rel}}}$. This constancy of $\overline {R{e}_{{\mathrm{eff}}}} $ possibly indicates a preferred Reynolds number for optimal lift generation by the wings of each species. Notably, $\overline {R{e}_{{\mathrm{eff}}}} $ relates to the relative velocity experienced by the wing, and thus to the quasi-steady aerodynamic load on the wing. Moreover, both insect species operate near important transitional points in unsteady aerodynamics, and their specialized wing kinematics may be most effective only within a narrow range of flow regimes. Therefore, neglecting nonlinear, complex unsteady loads, the animal might desire a constant aerodynamic load on the wings.

The specific patterns of how fruit flies and fungus gnats alter their wing kinematics provides insight into the relative importance of unsteady lift generation techniques for these two species, which occupy somewhat different Reynolds number regimes (${\mathrm{R}}{{\mathrm{e}}}_{\mathrm{w}} \approx 80$ and 50 for the fruit fly and fungus gnat, respectively). This is important because the stronger viscous forces at lower Reynolds numbers attenuate leading-edge vortex formation and drastically reduce the lift-to-drag ratio (Miller and Peskin 2004), thus forcing tiny insects to incorporate unsteady lift generation mechanisms such as the clap-and-fling (Sane 2003). Fruit flies generally only use the clap-and-fling mechanism when a rapid burst of lift is needed (e.g., during takeoff; Lehmann et al. 2005). We observed a reduced stroke amplitude and, in particular, a reduction in ${{\mathrm{\Phi }}}_{{\mathrm{max}}}$, in fruit flies in an updraft. This observation correlates with the lack of dorsal wing–wing interaction characteristic of the clap-and-fling mechanism (Fig. 4B) and would lead to reduced lift coefficient, which should also provide additional flight stability (Lehmann and Dickinson 2001; Ortega-Jiménez and Combes 2018) and conserved energy (Dickinson and Lighton 1995). In contrast, fungus gnats flying in the updraft retained a relatively similar ${{\mathrm{\Phi }}}_{{\mathrm{max}}}$ (i.e., in the dorsal position), thereby promoting the lift-generating clap-and-fling mechanism, while significantly truncating the stroke ventrally (Figs. 3A and B, and 4A). As fungus gnats operate at a lower $R{e}_{\mathrm{w}}$, they are more likely to rely heavily on this wing-wing interaction (i.e., clap and fling) to generate sufficient lift, while simultaneously conserving energy from the lower stroke amplitudes.

The updraft may also present a challenge to the insects’ flight stability. The smaller, slower flying fungus gnats had significantly reduced *η * (i.e., more sinuous trajectories) in the updraft, which may reflect the insects attempting to maintain stability in the turbulent flow. In a similar vein, fruit flies in a convective cell had more sinuous trajectories as compared to quiescent conditions, with the individuals failing to cross the cell having the greatest sinuosity (Ortega-Jiménez and Combes 2018). In contrast, the *η * values of the larger, faster flying fruit flies in our vertical wind tunnel were unaffected. These differences suggest that the updraft may have presented a greater threat to the flight stability of the smaller fungus gnat than to that of the fruit fly. Indeed, the scale of vortices relative to the size of the insect is thought to govern this threat (Ortega-Jiménez and Combes 2018). The length scale of turbulent eddies in our vertical wind tunnel is on the order of a millimeter. However, the fact that neither species altered beat frequency suggests that their flight stability was not overly threatened by the relatively small turbulent eddies and that the insects instead primarily focused on reducing lift to maintain elevation and save energy. Nonetheless, the flight exhibited by both species is remarkable as it shows a high degree of control even when exposed to large-scale atmospheric winds exceeding 200 body lengths per second.

The study had several limitations. First, the ambient illumination was not quantified. While efforts were made to ensure the room was as dark as possible, the experimental space had several sources of illumination that the insects may have detected. Further, the experiments were conducted during the day without regard to the circadian rhythm of the insects, which could affect their activity. In addition, we did not separate males and females. As a result, the males may have engaged in mate-searching behaviors that could have influenced flight trajectories. Additionally, the fruit flies were from a previously established culture, and long-term culturing is known to decrease animal’s flight performance and capabilities (Wheeler 2013). Furthermore, at present we only examined a single flow-case. Future experiments conducted at a range of speeds and turbulence intensities to mimic the more turbulent daytime updrafts within the CBL would also be useful.

## Conclusions

We used a vertical wind tunnel and a 3D photogrammetry system to examine the response of two insect species (fruit fly and fungus gnat) to a vertical flow meant to represent a nighttime updraft within the NBL. As in the field, both species maintained similar vertical velocities in both quiescent conditions and in an 0.433 m s^−1^ updraft by altering their wing and body kinematics. Both insect species achieved this by retaining a constant wingbeat frequency while reducing their stroke amplitude, though with a size-specific difference. Specifically, the smaller fungus gnats kept the dorsal portion of their stroke more so than the larger fruit flies, thereby maintaining the lift-generating clap-and-fling mechanism which is generally required at the lower Reynolds numbers at which they operate. For both species, the modulated wing kinematics resulted in similar relative flow velocity (and thus effective Reynolds number) across conditions, i.e. the combination of the wing tangential velocity and the animal velocity relative to the free stream. This similarity indicates that both species may have a preferred relative velocity for optimal lift generation. Finally, the updraft somewhat affected flight stability in the fungus gnat (as seen in its more sinuous flight trajectories) but not in the fruit fly, which did not change the sinuosity of its trajectories in the updraft. The observed decreases in body pitch angles in both species were likely linked to increases in animal speed. Overall, we conclude that these insect species resisted elevation changes from a vertical wind by actively altering wing kinematics to reduce their lift coefficient. Our findings thus provide insight into the small-scale behaviors underlying insect dispersal and migration, energy conservation mechanisms used by insects (which may also be employed by microaerial vehicles in the future), and establish a link between large-scale atmospheric observations and small-scale bio-locomotion analysis.

## Supplementary Material

icag140_Supplemental_File

## Data Availability

All data are available upon reasonable request.
