# Supplementary material for: Small insects actively respond to updrafts by modulating their wing and body kinematics
Source: Integr Comp Biol. 2026 Aug 3;66:icag140. doi: 10.1093/icb/icag140 (PMC13531471; doi:10.1093/icb/icag140)
Supplement: Supplementary file 1 [file icag140_supplemental_file.pdf]

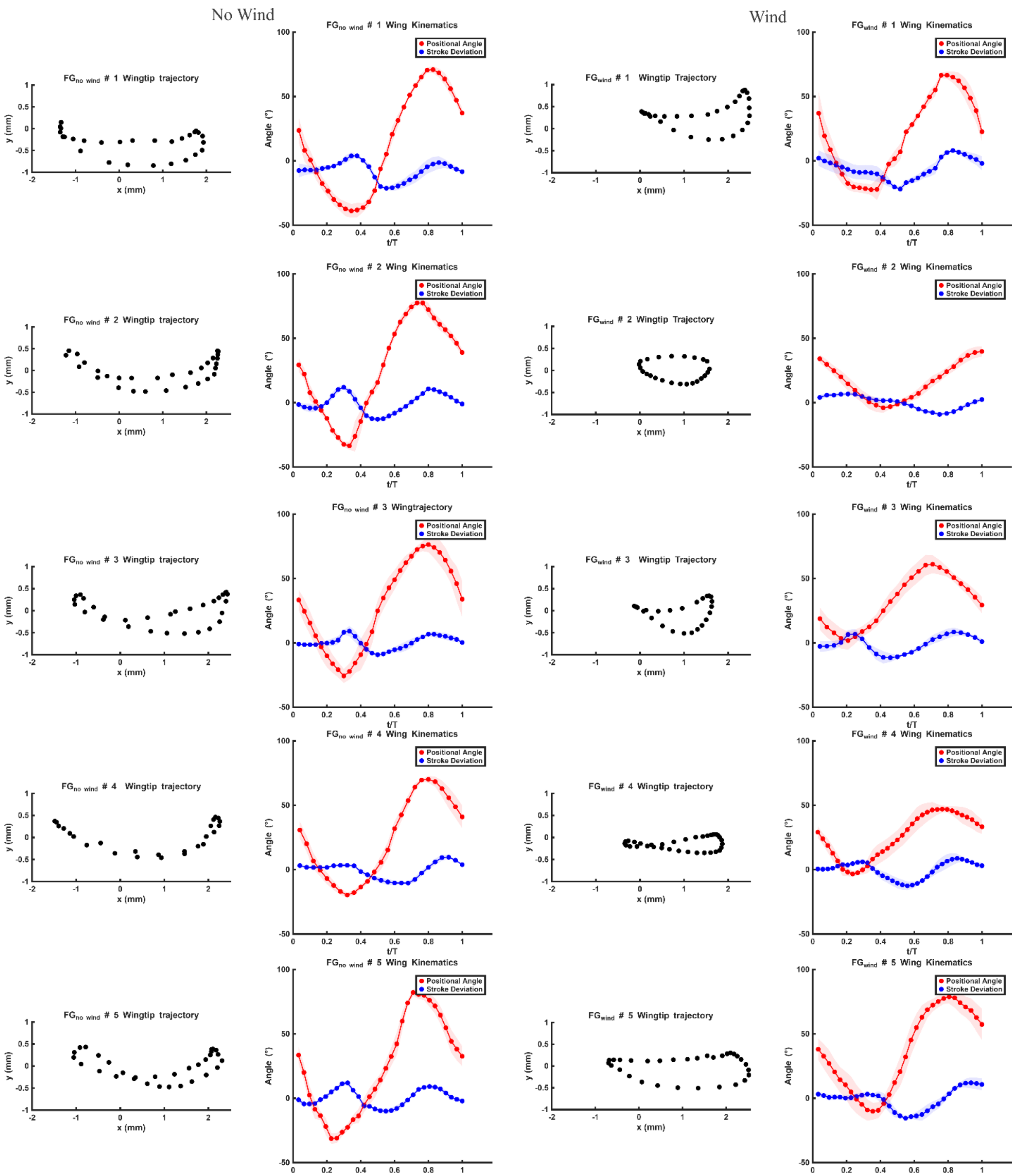

Figure SI 1: Average wing beat kinematics of individual fungus gnats across five wing consecutive wingbeats.

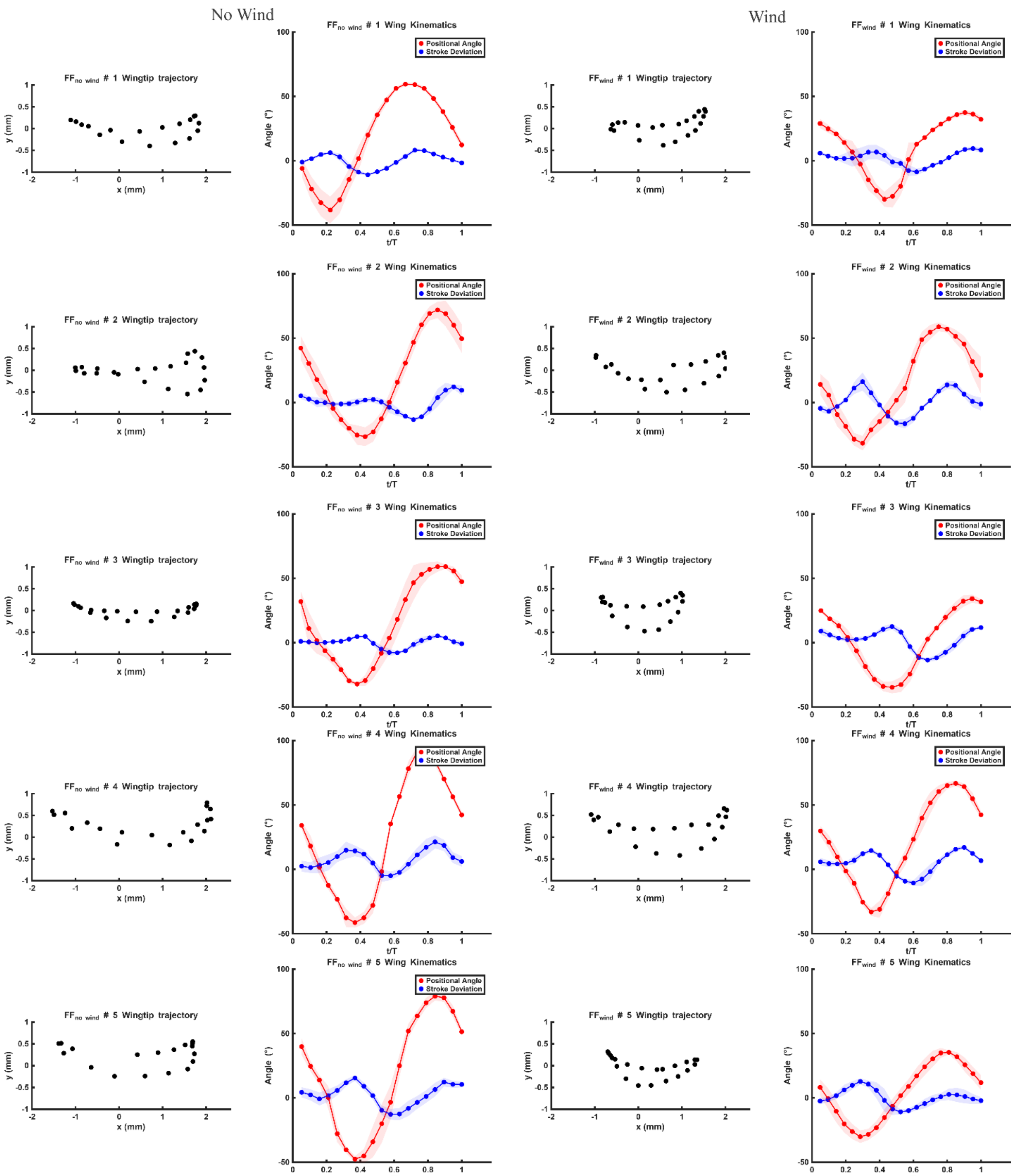

Figure SI 2: Average wing beat kinematics of individual fruit flies across five wing consecutive wingbeats.
